# Sequential treatment of progressive multifocal leukoencephalopathy with intravenous immunoglobulins and pembrolizumab

**DOI:** 10.1007/s13365-022-01059-2

**Published:** 2022-03-23

**Authors:** Fabian Boesl, Kristina Allers, Juliane Herm, Thomas Scheider, Christiana Franke

**Affiliations:** 1 Department of Neurology, Charité – Universitätsmedizin Berlin, corporate member of Freie Universität Berlin and Humboldt Universität Zu Berlin, 12200 Berlin, Germany; 2 Department of Gastroenterology, Infectious Diseases and Rheumatology, Charité – Universitätsmedizin Berlin, corporate member of Freie Universität Berlin and Humboldt Universität Zu Berlin, Berlin, Germany

**Keywords:** Progressive multifocal leukoencephalopathy, Intravenous immunoglobulins, Pembrolizumab, Rituximab, Follicular lymphoma

## Abstract

Progressive multifocal leukoencephalopathy (PML) is a rare demyelinating disease of the CNS caused by the human polyomavirus 2 (JCV). PML predominantly occurs in immunocompromised patients. To date, no specific antiviral treatment exists, leaving only restoration of the immune system as possible treatment. In 2019, the monoclonal antibody pembrolizumab was reported as a potential treatment option in PML in a case series. Following case reports could not thoroughly confirm a positive outcome. Pembrolizumab targets the inhibitory programmed cell death protein 1 (PD-1) receptor on lymphocytes and is associated with beneficial expansion of pre-existing virus-specific T cells. Here we describe a patient with PML who benefited from combined treatment with intravenous immunoglobulins, maraviroc, and pembrolizumab.

## Case presentation

**Fig. 1:**
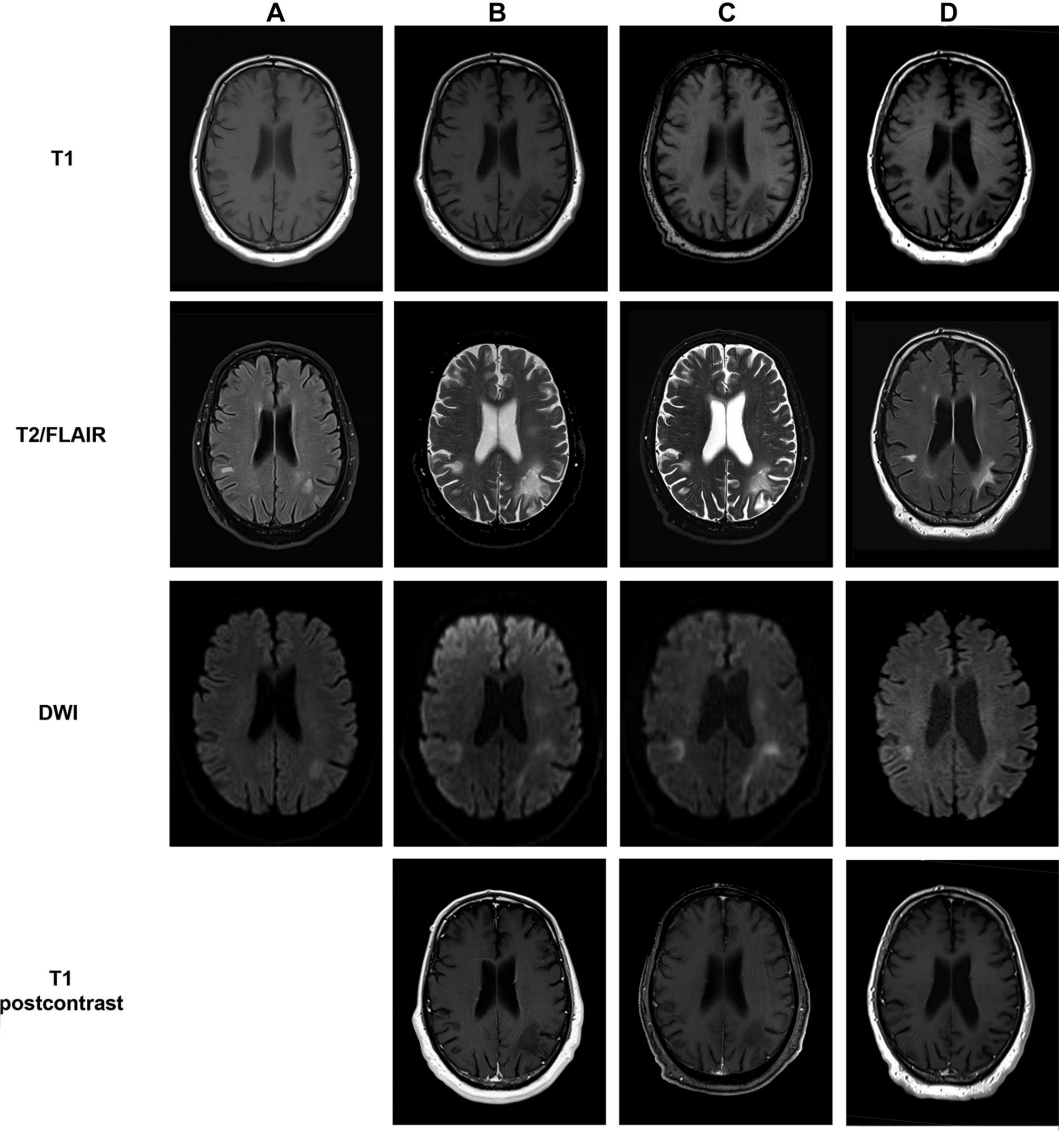
MRI of the head during treatment with pembrolizumab. Shown is a panel with sequential MRI scans of the head from July 2019 (**A**), before the third pembrolizumab infusion in January 2020 (**B**), the fifth pembrolizumab infusion in February 2020 (**C**), and after the final treatment with pembrolizumab in a follow-up examination in August 2020 (**D**). Shown sequences are T1 (**A**–**D**), T2-weighed (**B**–**C**) or FLAIR (**A**,** D**), DWI (**A**–**D**), and T1 postcontrast (**B**–**C**)

**Fig. 2:**
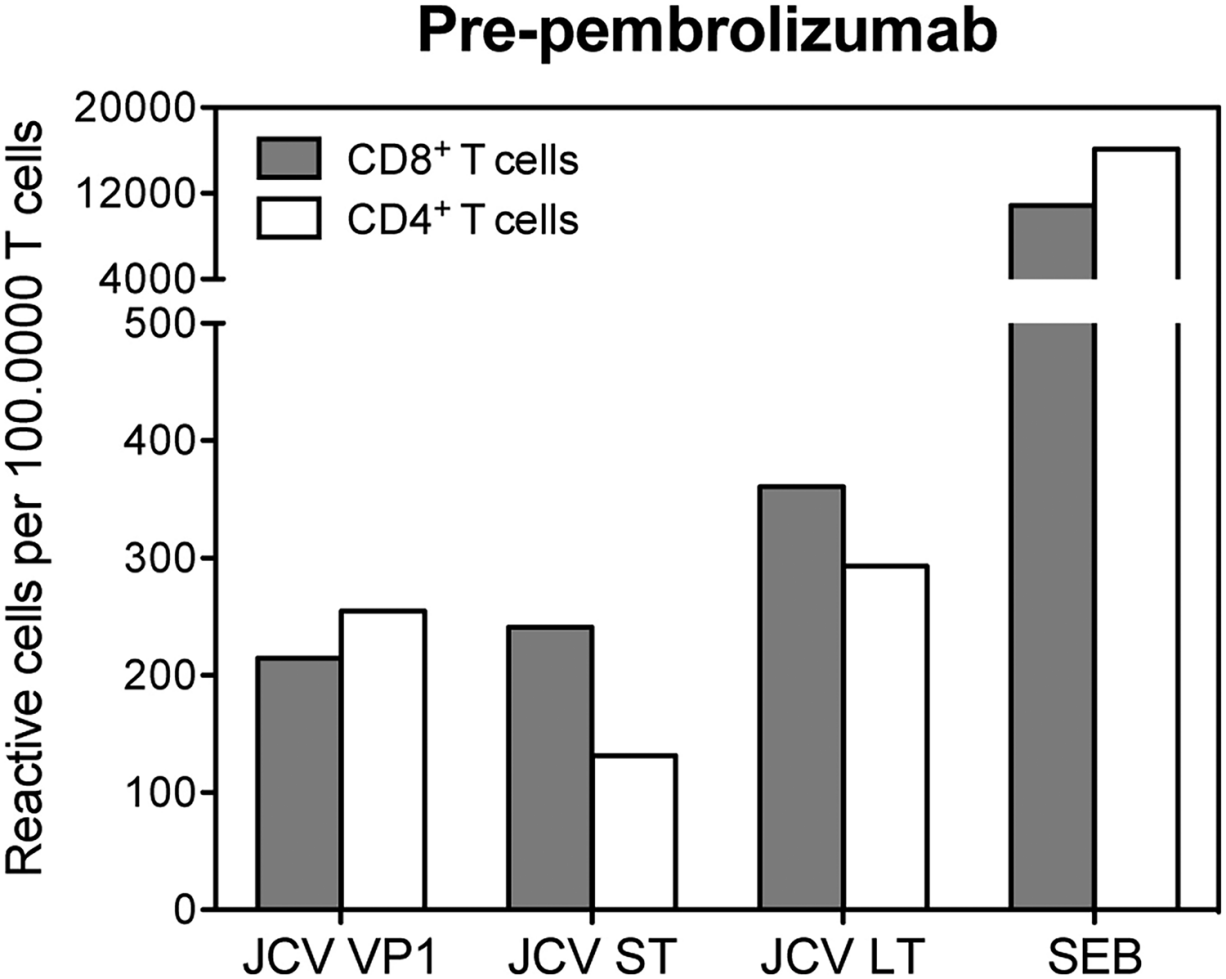
Frequencies of JC virus–specific CD4^+^ and CD8^+^ T cells. PBMC obtained before pembrolizumab treatment was stimulated with JC virus peptides covering the sequence of the capsid protein VP1, the small T antigen (ST), or the large T antigen (LT) for 5 h and analyzed for TNF production by flow cytometry. Stimulation with *Staphylococcus aureus* toxin B (SEB) served as positive control and incubation of cells without addition of stimulus served as background control

**Fig. 3:**
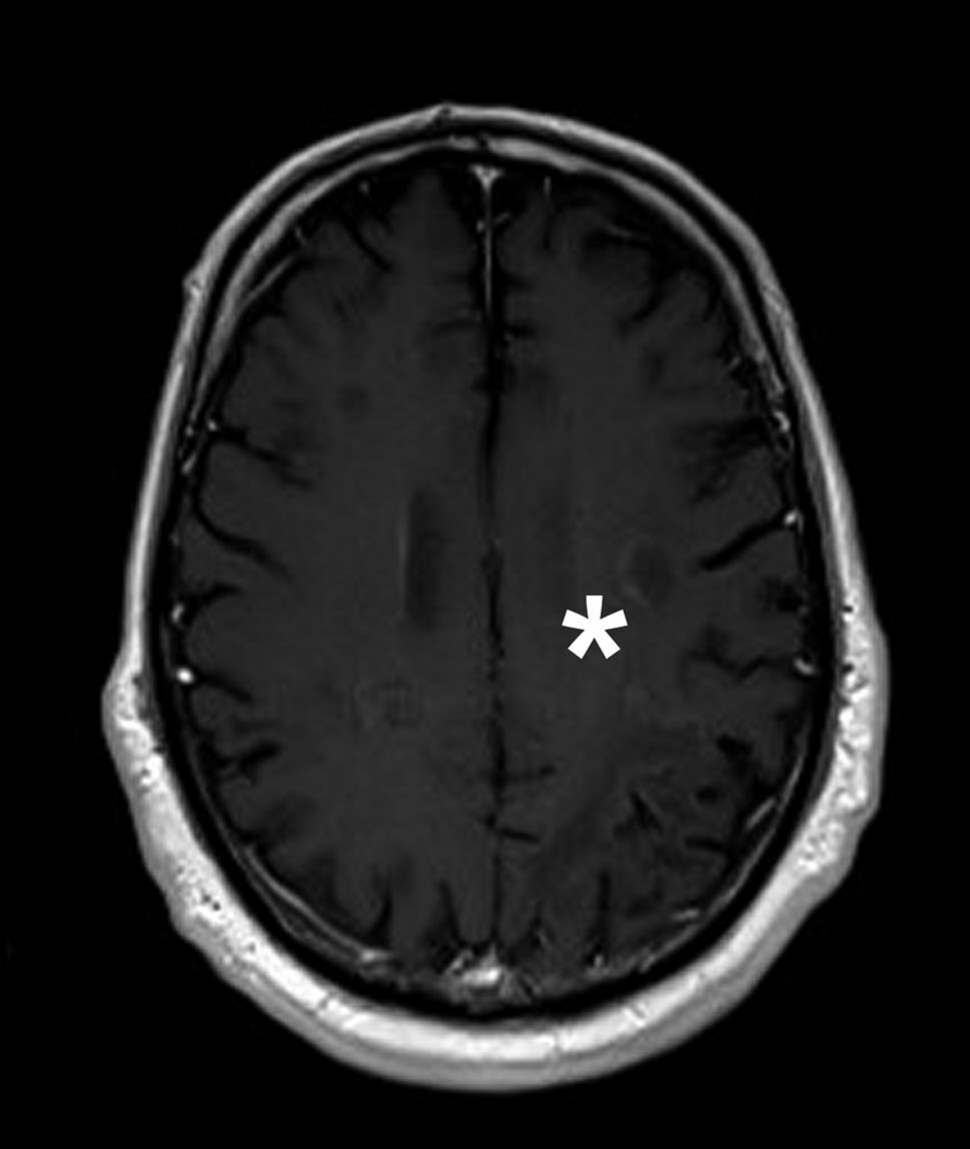
MRI of the head from January 2020 showing contrast enhancement. Shown is a T1 postcontrast MRI scan from January 2020. Slight gadolinium enhancement is marked with an asterisk (*)

In May 2019, a 69-year-old Caucasian presented in our outpatient clinic with double vision, gait ataxia, motor dysfunction of the right hand, amnestic deficits, and dysarthria. Treatment with rituximab was administered from April 2015 until January 2018 and R-bendamustine from April 2015 until October 2015 due to follicular lymphoma, diagnosed in March 2015. Progressive multifocal leukoencephalopathy (PML) was diagnosed in July 2019 due to typical cerebral MRI lesions (Fig. 1A) and positive human polyomavirus 2 (JCV) PCR in cerebrospinal fluid (CSF). Total T cell and CD4^+^ T cell count at that time was 325 and 214 per microliter of blood. JCV-specific cells were detectable in both CD4 + and CD8 + T cells (Fig. 2). As a result of long-term treatment with rituximab, a deficiency of IgM- (0.16 g/l), IgA- (0.78 g/l), and IgG antibodies (8.58 g/l) was noted. In December 2019, treatment with intravenous immunoglobulins (ivIg) (2 g/kg over 5 days) followed by 7 courses of pembrolizumab (2 mg/kg) was induced. Time between each dose of pembrolizumab was 3–4 weeks. To prevent an immune reconstitution inflammatory syndrome (IRIS), the patient received additionally maraviroc 600 mg/day, a CCR5 antagonist that seems beneficial in PML-IRIS (Hodecker et al. 2017). Regular neurological examination did not show any neurological deterioration but rather a robust improvement of the previously mentioned deficits that was already noticeable after the first pembrolizumab cycle. Neuropsychological assessments using the Montreal Cognitive Assessment Scale (MoCA) demonstrated a cognitive improvement from initially 7 to 17 of 30 points. Initial MRI scans of the head in July 2019 showed T2w lesions biparietal and in the right cerebellar hemisphere (Fig. 1A). A follow-up MRI before the third pembrolizumab infusion in January 2020 showed a progress of the lesions as well as new subcortical lesions frontal and temporal (Figs. 1B and 3). Second PCR testing for JCV DNA in CSF was weakly positive with a viral load between 500 and 2500 copies per microliter. In MRI follow-ups in February 2020 (Fig. 1C) and at the end of treatment (Fig. 1D), some lesions had receded. After the sixth infusion of pembrolizumab, JCV DNA returned undetectable in the patient’s CSF and frequencies of JC virus–specific T cells were lower compared to baseline. The total T cell count was restored to 1,213 per microliter of blood with 768 CD4^+^ T cells per microliter.

## Discussion

In this case, treatment with pembrolizumab might have supported pre-existing populations of JCV-specific T cells in fighting PML (Cortese et al. 2019; Tan et al. 2012), resulting in patient’s improvement. A case series published in 2019 showed a positive outcome of 5 of 8 patients with PML treated with pembrolizumab (Cortese et al. 2019). Combined with following case reports, until January 2021, 14 patients with PML due to lymphoproliferative disorders were treated with pembrolizumab (Cortese et al. 2019; Dufour et al. 2020; Holmes et al. 2020; Kapadia and Ney 2020; Mahler et al. 2020; Möhn et al. 2021; Rauer et al. 2019; Stögbauer et al. 2021). Of those, 9 patients stabilized or showed clinical improvement. Only 4 patients showed clinical or radiological signs of IRIS (Dufour et al. 2020; Möhn et al. 2021; Rauer et al. 2019). As IRIS is a possibly fatal complication in treating PML, we decided for additional treatment with maraviroc, a CCR5 antagonist that seems beneficial in PML-IRIS (Hodecker et al. 2017). Though there was slight gadolinium enhancement in an MRI during treatment (Fig. 3) as a possible subclinical sign of PML-IRIS (Wattjes et al. 2016), the patients’ symptoms did not deteriorate. No further contrast enhancement was detected in following MRI scans. To evaluate the effect of pembrolizumab in treating PML and the use of maraviroc in treating PML-IRIS, further studies with respect to the primary cause of immunodeficiency are warranted. Since prognosis of PML in patients with hematological malignancies is poor due to innate immunosuppression and respective immunomodulatory therapies (Neil and DeAngelis 2017), treatment of PML with pembrolizumab should be an option well considered.
